# Dietary Diversity and Undernutrition in Children Aged 6–23 Months in Sub-Saharan Africa

**DOI:** 10.3390/nu13103431

**Published:** 2021-09-28

**Authors:** Richard Gyan Aboagye, Abdul-Aziz Seidu, Bright Opoku Ahinkorah, Francis Arthur-Holmes, Abdul Cadri, Louis Kobina Dadzie, John Elvis Hagan, Oghenowede Eyawo, Sanni Yaya

**Affiliations:** 1 Department of Family and Community Health, School of Public Health, University of Health and Allied Sciences, Hohoe PMB 31, Ghana; raboagye18@sph.uhas.edu.gh; 2 Department of Population and Health, University of Cape Coast, Cape Coast TF0494, Ghana; abdul-aziz.seidu@stu.ucc.edu.gh (A.-A.S.); louis.dadzie1@stu.ucc.edu.gh (L.K.D.); 3 College of Public Health, Medical and Veterinary Sciences, James Cook University, Townsville, QLD 4811, Australia; 4 Department of Estate Management, Takoradi Technical University, Takoradi P.O. Box 256, Ghana; 5 School of Public Health, Faculty of Health, University of Technology Sydney, Sydney, NSW 2007, Australia; brightahinkorah@gmail.com; 6 Department of Sociology and Social Policy, Lingnan University, 8 Castle Peak Road, Tuen Mun, Hong Kong, China; frarthur88@gmail.com; 7 Department of Social and Behavioural Science, School of Public Health, University of Ghana, LG 25, Legon, Accra 23321, Ghana; abdul20c@yahoo.com; 8 Department of Family Medicine, Faculty of Medicine, McGill University, Montreal, QC H3S 1Z1, Canada; 9 Department of Health, Physical Education, and Recreation, University of Cape Coast, Cape Coast TF0494, Ghana; 10 Neurocognition and Action-Biomechanics-Research Group, Faculty of Psychology and Sport Sciences, Bielefeld University, Postfach 100131, 33501 Bielefeld, Germany; 11 School of Global Health, Faculty of Health, York University, 4700 Keele St, Toronto, ON M3J 1P3, Canada; oeyawo@yorku.ca; 12 School of International Development and Global Studies, University of Ottawa, 75 Laurier Ave. E, Ottawa, ON K1N 6N5, Canada; sanni.yaya@uottawa.ca; 13 The George Institute for Global Health, Imperial College London, 84 Wood Lane, London W12 0BZ, UK

**Keywords:** dietary diversity, stunting, wasting, underweight, undernutrition, sub-Saharan Africa

## Abstract

Dietary diversity plays a major role in the health status of children. However, evidence on its crucial role on children’s health status remains inconclusive in sub-Saharan Africa (SSA). In this study, we examined the association between dietary diversity and undernutrition among children aged 6–23 months in SSA. We pooled data from the most recent Demographic and Health Surveys of 32 countries in SSA from 2010 to 2020. A sample of 48,968 mother-child pairs of children within the ages of 6–23 months and mothers aged 15–49 years were included in this study. Multilevel logistic regression analysis was carried out to examine the association between dietary diversity and stunting, wasting, and underweight. The results were presented as crude odds ratios (cOR) and adjusted odds ratios (aOR) with their 95% confidence intervals. Statistical significance was set at *p* < 0.05. The overall prevalence of minimum dietary diversity was 25.1%, with South Africa recording the highest prevalence (43.9%) and Burkina Faso recording the lowest prevalence (5.6%). The highest prevalence of stunting was recorded by Burundi (51.8%) while the lowest prevalence was found in Ghana (13.6%), with an overall regional prevalence of 28.6%. For wasting, prevalence from all countries was found to be 9.4%. South Africa recorded the lowest prevalence of wasting (2.1%) while Niger recorded the highest prevalence (27.3%). Lastly, the prevalence of underweight ranged from 5.3% in South Africa to 41.8% in Niger, with an all-country prevalence of 16.4%. Children who had adequate minimum dietary diversity had 12% less likelihood of being stunted (aOR = 0.88, 95% CI = 0.83, 0.94), compared to those who had inadequate minimum dietary diversity. Having an adequate minimum dietary diversity significantly lowered the risk of underweight among children by 17% (aOR = 0.83, 95% CI = 0.77, 0.91). Having an adequate minimum dietary diversity was associated with 13% reduced odds of wasting among children (aOR = 0.87, 95% CI = 0.78, 0.97), compared to those who had inadequate minimum dietary diversity. This study highlights the significant association between minimum dietary diversity and stunting, wasting, and underweight among 6–23 month-old children in SSA. There is an urgent need for additional nutrition-specific interventions and strengthening of existing interventions aimed at improving infant and young child feeding practices, including complementary feeding practices among children aged 6–23 months in the 32 countries in SSA. Such interventions should focus more on countries where the prevalence of adequate minimum dietary diversity was low and undernutrition was high.

## 1. Introduction

Nutrition is an essential component of the Sustainable Development Goal (SDG) 2 which focuses on zero hunger by 2030 [1]. However, many parents, particularly those in low-and middle-income countries struggle to provide dietary diversity for children [2]. Dietary diversity is considered to be an outcome of the nutritional status of children [3,4] and a key component of high-quality diets [5,6,7]. Dietary diversity is used as a tool to measure dietary quality, micronutrient adequacy, and food access [8,9,10,11] and it influences the health outcomes of children under five [3,12]. Studies show that inadequate dietary quality and diversity lead to undernutrition including stunting, underweight, and wasting [4,13]. The World Health Organization (WHO) recommends that young children, especially infants should receive a minimum of four out of the seven groups of foods-namely: grains, roots, and tubers; legumes and nuts; dairy products; flesh foods (meat, fish, poultry, and organ meats); eggs; vitamin-A—rich fruits and vegetables; and other fruits and vegetables [5,14,15]—to maintain proper growth and development [15]. Yet, many children in low- and middle-income countries do not have adequate dietary diversity [8,9,10,11]. Less than one-fourth of children aged 6–23 months met the minimum acceptable diet dietary diversity and meal frequency standards in low and middle-income countries [16].

Undernutrition is regarded as the most prevalent form of malnutrition with its indicators being stunting, wasting, and underweight [17]. Evidence suggests that undernutrition is associated with increased childhood morbidities and mortalities [17]. According to the WHO, 149 million children under five years are stunted and 45 million are wasted worldwide [17]. In terms of regional analysis, 57.5 million, 11.8 million, 3.0 million children under five years respectively were stunted, wasted, and severely wasted in 2019 [18]. Achieving the minimum dietary diversity (MDD) for children under five in sub-Saharan Africa (SSA) is a big challenge due to poverty which is related to the low-income status of many parents as they struggle to provide better complementary feeding practices for babies or meet the minimum standards of dietary diversity for infants and young children [8,9]. Household food insecurity has been identified as a factor for inadequate dietary diversity for infants in most sub-Saharan African countries [19,20,21,22].

Families with higher incomes have more diverse diets, which resulting in positive nutritional status of infants and young children [23,24]. Studies have shown that dietary diversity is associated with the nutritional status of infants and children [12,25,26]. For instance, a study conducted in Indonesia found that individual dietary diversity is strongly associated with stunting in infants and young children [25]. In Nigeria, a study also showed that only 31.5% of children met the MDD and 23.1% met the minimum acceptable diets [27]. In Ethiopia, Eshete and colleagues [24] found that inadequate MDD among children aged 6–23 months was high (85.1%). However, to date, empirical findings explaining the association between dietary diversity and nutritional status among children in SSA remains inconclusive. This study, therefore, seeks to fill this research gap by examining the prevalence of dietary diversity and undernutrition among children in SSA as well as the association between the two variables. This study provides insights into the links between dietary diversity and nutritional status of children, particularly those aged 6 to 23 months for interventions to reduce undernutrition among infants and young children in the SSA.

## 2. Materials and Methods

### 2.1. Data Source and Study Design

Data for this study were obtained from the recent Demographic and Health Surveys (DHS) of 32 sub-Saharan African countries for the period between 2010 and 2020. The DHS is a nationally representative survey conducted in over 90 low- and middle-income countries globally. The DHS adopted a cross-sectional design relying on a two-stage stratified sampling technique to recruit its respondents [28]. The survey focuses on essential maternal and child health markers and men’s health. The request for the use of the data set was made through Measure DHS and permission was granted. The dataset is freely accessible via this link: https://dhsprogram.com/data/available-datasets.cfm (accessed on 1 August 2021). A sample of 48,968 mother-child pairs of children within the ages of 6–23 months and mothers aged 15–49 years were included in this study (Table 1). All children with valid anthropometric measurements were included.

### 2.2. Study Variables

#### 2.2.1. Outcome Variables

The outcome variables in this study were the three indicators used to measure undernutrition. These indicators include stunting, wasting, and underweight. To assess the outcome variables, we used child growth failure, which was classified with respect to height-for-age z-score (HAZ), weight-for-age z-score (WAZ), and weight-for-height z-score (WHZ) as defined by the WHO Growth reference standard [29]. On the basis ofthe WHO Growth reference standard, any child, which in this study was limited to those aged 6–23 months, whose HAZ, WAZ, and WHZ fell below minus 2 (−2.0) standard deviations (SD) less than the mean on the reference standard for a given age was classified as stunted, underweight, and wasted respectively. Those who were stunted, underweight, and wasted were coded as “1” while those normal were coded as “0”.

#### 2.2.2. Explanatory Variables

MDD, an indicator that measures dietary diversity was the key explanatory variable. MDDcan be defined as the proportion of children aged 6–23 months who receive four or more out of seven food groups [4,12,13,15,30]. The children were expected to consume at least four of the seven food groups in addition to breastmilk. The seven food groups included (i) grains, roots, and tubers; (ii) legumes and nuts; (iii) dairy products (milk, yogurt, cheese); (iv) flesh foods (meat, fish, poultry, liver, or other organs); (v) eggs; (vi) vitamin A-rich fruits and vegetables; and (vii) other fruits and vegetables. To estimate MDD, we summed all the food groups together with scores ranging from 0 to 7. Any child who consumed any of the food groups was assigned a score of one (1) and zero (0) for not consuming a food group. The children who consumed at least four (≥4) of the food groups were said to have an adequate MDD and this was coded as “1” while the remaining children who consumed lower than four food groups were coded as “0 = inadequate”. This classification and categorization were informed by literature that studied either dietary diversity alone or its association with undernutrition [4,12,13,30].

Other explanatory variables were included as covariates. These variables were selected on the basis of their significant relationship with childhood undernutrition from literature [30,31,32,33,34] as well as their availability in the DHS dataset. The variables were grouped into individual-level, household-level, and contextual-level variables. The individual-level variables consisted of the characteristics of the child and the mother: sex of child, age of child, birth order, size of child at birth, mother’s age, maternal educational level, current working status, number of antenatal care attendance (ANC), place of delivery, postnatal care attendance (PNC), and marital status. Household-level variables include drinking water source, toilet facility, household size, frequency of watching television, frequency of listening to radio, frequency of reading newspaper/magazine, cooking fuel, and wealth index. The contextual-level variables comprised of place of residence, and geographical sub-regions (Southern, Central, Eastern, Western).

### 2.3. Data Preparation and Statistical Analyses

Prior to performing the statistical analyses, we checked for missing observations and any small cell frequencies through cross-tabulation between the explanatory and outcome variables. Categories deemed to have small cell frequencies were merged in ways that preserved common-sense meanings for each category. We included sampling weight (v005/1,000,000), clustering, and stratification variables which were provided by DHS to account for the complex survey design. The Stata command ‘*svyset*’ was used to declare the survey design, while all estimations were performed by using survey-specific command ‘*svy*.’ Map and forest plots were used to summarize the prevalence of MDD, stunting, wasting, and underweight. We performed a cross-tabulation to examine the distribution of the outcome variables across the explanatory variables. A univariable Pearson chi-square analysis was fitted with each explanatory variable and each outcome variable with the results presented using *p*-values. Due to the numerous covariates studied, the best subset variable selection method by Lawless and Singhal [35] was employed using the Stata command ‘gvselect’ to select variables for the next stage of the analysis which involvef a multivel binary logistic regression. This technique allows for the selection of a set of variables that best explain the outcome variable. We used three models under the multilevel regression analysis to establish the association between MDD and stunting, wasting, and underweight [35,36]. 

The first model (Model 0) was fitted to show the variance in the outcome variables attributed to the clustering of the primary sampling units (PSUs) and the explanatory variables. Model I was fitted to include only the key explanatory variables and the outcome variables. Model II was fitted to include all the explanatory variables against each of the outcome variables. We used AIC to test for model fitness and model comparison. The results of the regression analysis were presented using crude odds ratios (cOR) adjusted odds ratios (aOR) with their 95% confidence intervals (CIs). Statistical significance was set at *p* < 0.05. Stata software version 16.0 (Stata Corporation, College Station, TX, USA) was used to perform the analysis. We also drafted this manuscript relying on the Strengthening Reporting of Observational Studies in Epidemiology (STROBE) reporting guidelines [37].

## 3. Results

### 3.1. Prevalence of Minimum Dietary Diversity, Stunting, Wasting, and Underweight

**Figure 1:**
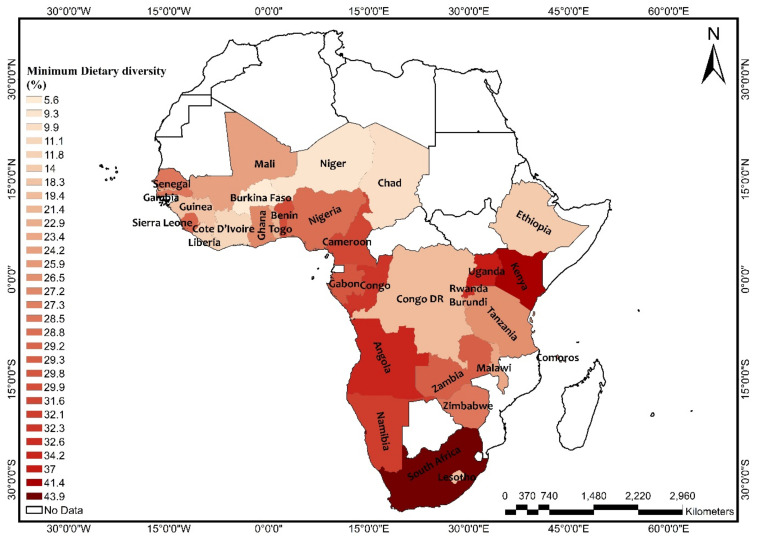
Map showing the prevalence of adequate minimum dietary diversity in sub-Saharan Africa. Source: Constructed on the basis of shapefiles from https://tapiquen-sig.jimdofree.com/descargas-gratuitas/mundo/ (accessed on 1 August 2021) with permission from Carlos Efrain Porto Tapiquen, 2021.

**Figure 2:**
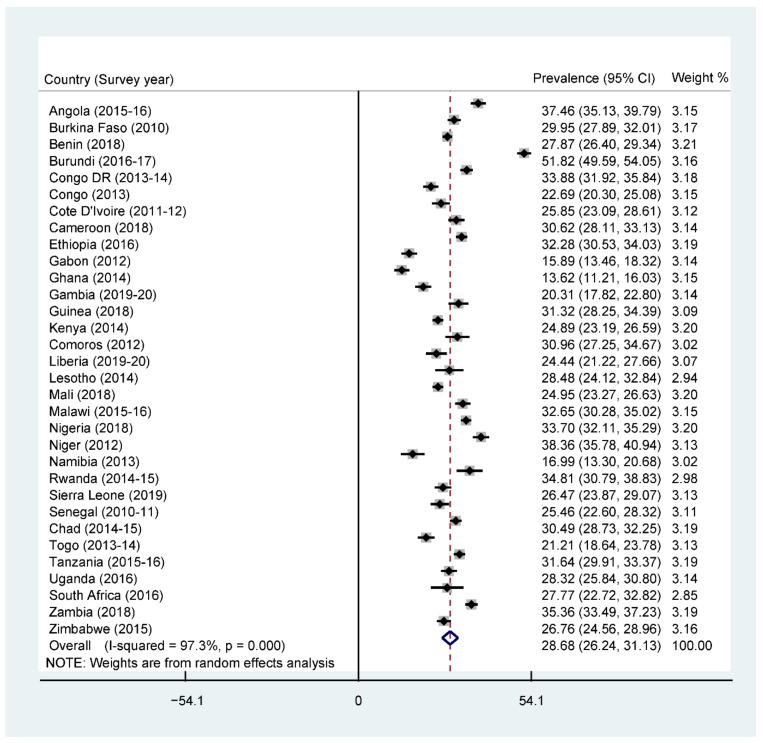
Forest plot showing the prevalence of stunting in sub-Saharan Africa.

**Figure 3:**
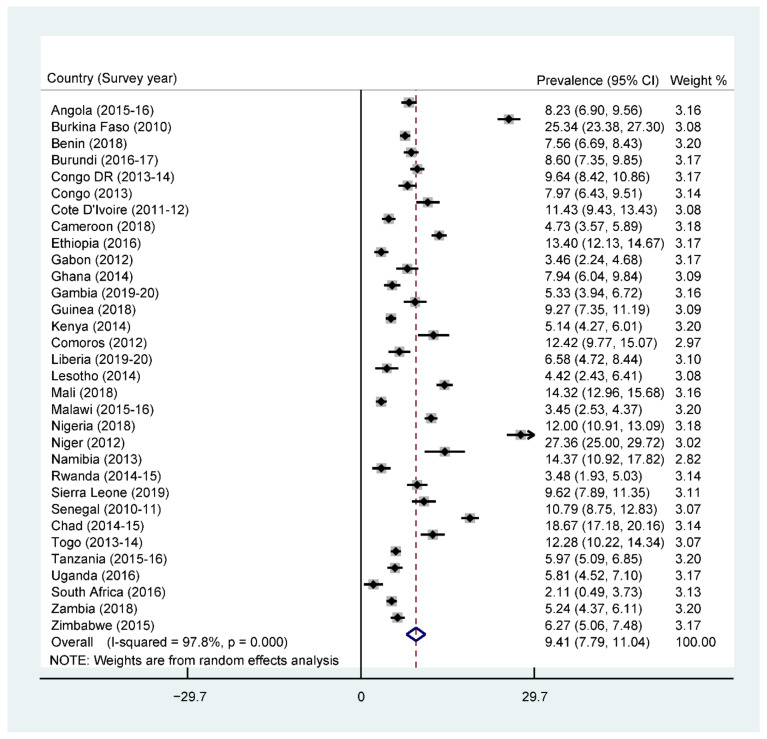
Forest plot showing the prevalence of wasting in sub-Saharan Africa.

**Figure 4:**
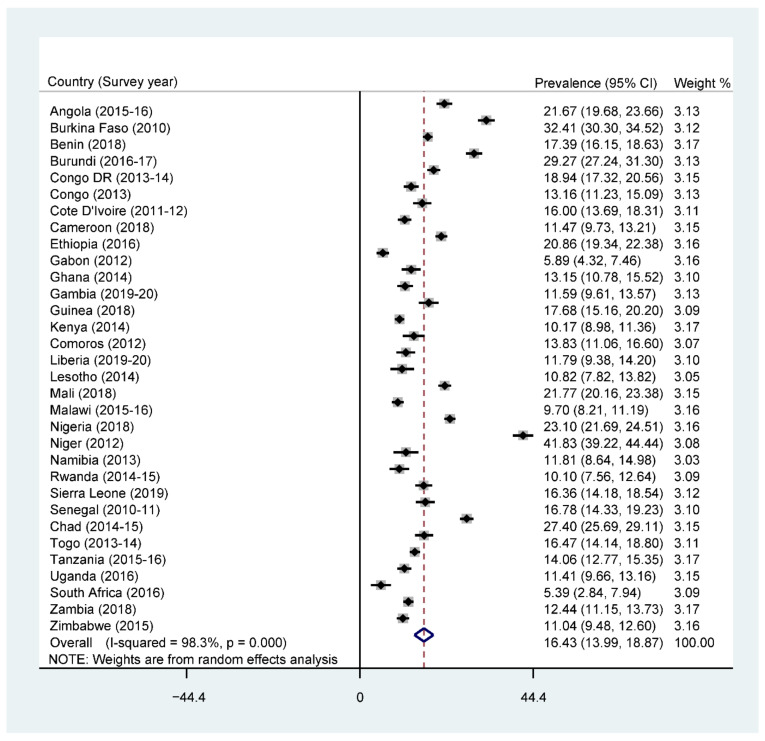
Forest plot showing the prevalence of underweight in sub-Saharan Africa.

The overall prevalence of adequate MDD was 25.1% across SSA, with South Africa recording the highest prevalence (43.9%) and Burkina Faso recording the lowest prevalence (5.6%) (Figure 1). Further results showed that 76% of the children were given grains, roots, and tubers. The consumption of grains, roots, and tubers was highest in Zimbabwe (94.4%) and lowest in Benin (54.3%) (See Supplementary Table S1). The highest prevalence of stunting was recorded by Burundi (51.8%), while the lowest prevalence was found in Ghana (13.6%), with an overall regional prevalence of 28.6% (Figure 2). For wasting, prevalence from all countries was found to be 9.4%. South Africa recorded the lowest prevalence of wasting (2.1%) while Niger recorded the highest prevalence (27.3%) (Figure 3). Lastly, the prevalence of underweight ranged from 41.8% in Niger to 5.3% in South Africa, with an all-country prevalence of 16.4% (Figure 4).

### 3.2. Bivariate Analysis of Minimum Dietary Diversity and Undernutrition among Children in SSA

Table 2 presents the bivariate analysis of dietary diversity and undernutrition among children in SSA. The results showed that 30.9% of the children who had inadequate dietary diversity were stunted. Additionally, 19.6% of the children who had inadequate dietary diversity were underweight whereas 11% of them were wasted. For the child characteristics, male children were found to be more stunted (34.0%), underweight (20.7%), and wasted (11.6%) compared to female children. The results on the maternal characteristics showed that the highest prevalence of stunting was found in children whose mothers were in the age range 45–49 (34.8%). For underweight, the highest prevalence was found in children whose mothers were in the age range 40–44 (22%), and then again, children whose mothers were in the age range 40–44 recorded the highest prevalence of wasting (11.9%). Mothers with no formal education recorded the highest prevalence of children who were stunted, underweight, and wasted, compared to those with some level of education. Under household level factors, children who drank from unimproved water source recorded higher prevalences of stunting (31.4%), underweight (19.5%), and wasting (10.9%), compared to those who drank from an improved water source. Children born in rural areas were found to be more stunted (33.6%), underweight (20.9%) and wasted (11.2%), compared to those in urban areas.

### 3.3. Association between Minimum Dietary Diversity and Stunting

The results on the association between MDD and stunting are presented in Table 3. The results of the complete model (model II—including all explanatory variables) show that children who had adequate MDD had 12% less likelihood of being stunted (aOR = 0.88, 95% CI = 0.83, 0.94), compared to those who had inadequate MDD. It was found that children who were smaller at birth were two times as likely to be stunted (aOR = 2.08, 95% CI = 1.93, 2.25) as compared to those who were large at birth. Additionally, an increasing number of ANC attendance significantly reduced the odds of stunting, with mothers who had 4 or more ANC attendance presenting 20% reduced odds (aOR = 0.80, 95% CI = 0.73, 0.88) of having a stunted child. Similarly, mothers who attended PNC had a 9% reduced likelihood (aOR = 0.91, 95% CI = 0.87, 0.96) of having a stunted child, compared to those who did not attend PNC. Moreover, children who belonged to large household sizes had a 14% increased odds of being stunted (aOR = 1.14, 95% CI = 1.05, 1.25) compared to those from a small household.

### 3.4. Association between Minimum Dietary Diversity and Underweight

The results of the complete model (model II) on the association between dietary diversity and underweight show that having an adequate MDD lowered the risk of underweight among children by 17% (aOR = 0.83, 95% CI = 0.77, 0.91). Additionally, children whose size was smaller at birth were twice as likely to be underweight (aOR = 2.72, 95% CI = 2.48, 2.98), compared to those whose size were large at birth. Moreover, children with an unimproved toilet facility had an increased likelihood of being underweight (aOR = 1.10, 95% CI = 1.03, 1.18), as well as those who were located in rural areas (aOR = 1.15, 95% CI = 1.05, 1.25). It was also found that increased frequency of a mother watching television presented a reduced likelihood of having an underweight child, with mothers who watched television more than once in a week presenting a 23% reduced likelihood of having an underweight child (aOR = 0.77, 95% CI = 0.70, 0.85). The results are presented in Table 4.

### 3.5. Association between Minimum Dietary Diversity and Wasting

Table 5 presents the results of the association between dietary diversity and wasting. The complete model (model II) shows again that, having an adequate MDD presented 13% reduced odds of wasting among children (aOR = 0.87, 95% CI = 0.78, 0.97), compared to those who had inadequate MDD. Additionally, birth order 5 and above increased the risk of wasting (aOR = 1.15, 95% CI = 1.02, 1.30) in children. Moreover, children whose size was smaller at birth were twice as likely to be wasted (aOR = 2.03, 95% CI = 1.83, 2.25) compared to those who had a larger size at birth. Having an unimproved toilet facility in the house also presented an increased likelihood of wasting (aOR = 1.14, 95% CI = 1.04, 1.25) compared to those who had an improved toilet facility. A mother attending ANC 4 or more times also presented odds22% reduced odds of wasting in children (aOR = 0.78, 95% CI = 0.70, 0.88).

## 4. Discussion

This study examined the association between dietary diversity and undernutrition (stunting, wasting, and underweight). The association between other child and maternal characteristics, household, and contextual factors and undernutrition was also examined. The study found a significant association between dietary diversity and stunting; in particular, that having adequate dietary diversity reduced the likelihood of stunting among children. Moreover, the study found a significant association between dietary diversity and underweight; that is, an adequately diversified diet reduced the likelihood of being underweight. Lastly, the study found that dietary diversity was significantly associated with wasting, emphasizing that adequate dietary diversity significantly reduced the odds of wasting among children in SSA.

The findings on the association between MDD and underweight is similar to findings by other studies from Uganda [38], South Africa [39], Nigeria [40], and Kenya [41]. A major factor that explains this finding is similar to the explanation above, where adequate dietary diversity means receiving foods from a minimum of four of the seven foods groups [5,14], which has been posited by Walingo and Ekesa [42] to reduce the risk of underweight among children. Additionally, Hooshmand and Udipi [43] reported that the nutritional status of children is influenced by their diet and an increase in consumption of diversified foods could reduce undernutrition including underweight among children.

Similarly, an association between MDD and stunting was also established, a finding that is consistent with findings by another study in SSA [44]. Previous studies conducted in Ethiopia [21], Nigeria [45], Ghana [13], South Africa [39], and Burkina Faso [46] also found that adequate dietary diversity significantly reduced the odds of stunting among children. Several factors could explain this finding. A major factor for explaining this finding is that, when children have an adequate dietary diversity, it means they receive foods from a minimum of four of the seven groups of foods- namely grains, roots, and tubers; legumes and nuts; dairy products; flesh foods (meat, fish, poultry, and organ meats); eggs; vitamin- A rich fruits and vegetables; other fruits and vegetables [5,14]. Receiving foods from these groups provides children with sufficient nutrients needed to reduce the risk of stunting [47] as found in this study.

Findings from other studies in South Ethiopia [48], South Africa [39], and Burundi and Democratic Republic Congo [49] mirror the observed association in this study. Importantly, adequate dietary diversity is receiving foods from a minimum of four of the seven groups of foods [5,14] and this will provide children with the sufficient nutrients needed to reduce the likelihood of wasting among them [13]. However, the findings from this study on the association between adequate MDD and wasting was incongruent with findings from another study in Burkina Faso [46]. Sié et al. [46] found that dietary diversity was not associated with wasting among children. The difference in the findings could be due to the different methods used, as the study by Sié et al. [46] was a randomized control trial and included children aged 6–59 months, whereas the present study is a secondary data analysis of cross-sectional DHS data from several SSA countries.

Other results indicate that the size of a child at birth increased their likelihood of undernutrition (stunting, wasting, and underweight). Children whose size was smaller at birth had higher odds of becoming malnourished, compared to those whose size was large. The findings of this study on the association between the size of a child at birth and undernutrition is supported by other studies [50,51]. In support of the findings of this study are findings of another study by Kumar, Abbas, Mahmood, and Somrongthong [52]. This study also found that babies born with small size are at increased likelihood of being underweight, compared to those with a large size at birth. With respect to the size of a child at birth and wasting, another study by Akombi et al. [32] found that children with small birth sizes had an increased likelihood of wasting, as compared to those with large birth sizes, which is similar to the findings in the present study. Similarly, Akombi et al. [32] found again that children born with small size and increased odds of stunting which corroborates the findings in the present study.

### Strengths and Limitations

The study has some limitations. First, since the study relied on secondary data, the analysis was limited to only variables that were in the dataset. Hence, interpretations and inferences made from our study should be considered in light of the included variables only. Additionally, the DHS employs cross-sectional designs, which restrict the analysis of causality on the noted outcomes. The key variables were self-reported by the mothers, and therefore, there is the likelihood of recall bias and other social desirability concerns. Additionally, our study did not adjust for total calories due to the non-availability of data on calorie consumption from the DHS and this could affect the extrapolations from this study. Despite the limitations, the study has strengths. The use of nationally representative data and a large sample size is a major strength of the study. Moreover, the findings from the study contribute to addressing the gap in literature on dietary diversity and undernutrition in SSA.

## 5. Conclusions

The findings suggest, that there is significant association between dietary diversity and stunting, wasting, and underweight among 6–23 month-old children in SSA. Other key covariates that are significantly associated with these undernutrition indicators were also identified. Therefore, there is a need for further nutrition-specific interventions and strengthening existing interventions aimed at improving infant and young child feeding (IYCF) practices, including complementary feeding practices among children aged 6–23 months in the 32 countries in SSA. These interventions should include evidence-based education models on the right feeding practices for childbearing women, as well as addressing the barriers to adequate feeding practices. Such interventions should be more focused on countries where the prevalence of minimum dietary diversity is low and undernutrition is high. Future studies should examine the effect of the specific food groups included in dietary diversity on the nutritional status of children.

## Figures and Tables

**Table 1:** Description of the study sample.

Countries	Year of Survey	Weighted N	Weighted %
Angola	2015–16	1652	3.4
Burkina Faso	2010	1894	3.9
Benin	2018	3576	7.3
Burundi	2016–17	1925	3.9
Congo DR	2013–14	2251	4.6
Congo	2013	1182	2.4
Cote d’Ivoire	2011–12	970	2.0
Cameroon	2018	1292	2.6
Ethiopia	2016	2754	5.6
Gabon	2012	867	1.8
Ghana	2014	781	1.6
Gambia	2019–20	1005	2.0
Guinea	2018	878	1.8
Kenya	2014	2474	5.0
Comoros	2012	595	1.2
Liberia	2019–20	686	1.4
Lesotho	2014	411	0.8
Mali	2018	2537	5.2
Malawi	2015–16	1,507	3.1
Nigeria	2018	3,411	7.0
Niger	2012	1,369	2.8
Namibia	2013	398	0.8
Rwanda	2014–15	539	1.1
Sierra Leone	2019	1110	2.3
Senegal	2010–11	892	1.8
Chad	2014–15	2627	5.4
Togo	2013–14	974	2.0
Tanzania	2015–16	2778	5.7
Uganda	2016	1269	2.6
South Africa	2016	302	0.6
Zambia	2018	2513	5.1
Zimbabwe	2015	1549	3.2
**All countries**		**48,968**	**100.0**

**Table 2:** Bivariate analysis of minimum dietary diversity and undernutrition among children aged 6–23 months in sub-Saharan Africa.

Variables	Weighted N	Weighted %	Stunted	*p*-Value	Underweight	*p*-Value	Wasted	*p*-Value
**Minimum dietary diversity**				<0.001		<0.001		<0.001
Inadequate	36,930	75.4	30.9		19.6		11.0	
Adequate	12,038	24.6	27.7		13.9		7.5	
**Child characteristics**								
**Sex of child**				<0.001		<0.001		<0.001
Male	24,663	50.4	34.0		20.7		11.6	
Female	24,305	49.6	26.2		15.6		8.6	
**Age of child**				<0.001		<0.001		<0.001
6–8	8953	18.3	16.7		14.3		11.2	
9–11	8328	17.0	21.9		17.6		12.0	
12–17	17,471	35.7	31.5		18.9		10.4	
18–23	14,216	29.0	41.8		20.1		8.1	
**Birth order**				<0.001		<0.001		<0.001
1	10,423	21.3	29.4		15.7		8.5	
2–4	23,569	48.1	28.9		16.9		9.5	
5 and above	14,976	30.6	32.6		22.0		12.3	
**Size of child at birth**				<0.001		<0.001		<0.001
Large	16,972	34.7	24.7		13.5		8.1	
Average	24,209	49.4	30.9		17.9		9.9	
Smaller	7787	15.9	39.8		29.4		15.2	
**Maternal characteristics**								
**Mother’s age**				<0.001		<0.001		<0.001
15–19	4428	9.0	32.1		17.6		10.1	
20–24	12,087	24.7	31.2		17.3		9.1	
25–29	13,302	27.2	29.0		17.6		9.8	
30–34	9684	19.8	28.5		18.7		10.6	
35–39	6417	13.1	30.1		19.0		11.4	
40–44	2491	5.1	33.4		22.0		11.9	
45–49	560	1.1	34.8		19.5		11.6	
**Maternal educational level**				<0.001		<0.001		<0.001
No education	18,997	38.8	34.9		25.7		14.5	
Primary	16,054	32.8	31.8		16.0		8.0	
Secondary	12,387	25.3	23.0		10.9		6.7	
Higher	1530	3.1	11.7		6.2		5.3	
**Current working status**				0.033		0.141		<0.001
No	18,720	38.2	29.4		18.6		11.3	
Yes	30,248	61.8	30.6		17.9		9.4	
**ANC**				<0.001		<0.001		<0.001
None	5046	10.3	38.1		28.6		16.4	
1–3	16,750	34.2	33.7		20.5		11.2	
4 or more	27,172	55.5	26.5		14.9		8.3	
**Place of delivery**				<0.001		<0.001		<0.001
Home	14,923	30.5	36.1		25.9		14.4	
Health facility	33,472	68.4	27.4		14.7		8.3	
Other	567	1.1	36.4		20.6		7.3	
**PNC**				<0.001		<0.001		0.003
No	28,940	59.1	31.9		19.5		10.6	
Yes	20,028	40.9	27.6		16.3		9.5	
**Marital status**				0.004		<0.001		<0.001
Never in union	3411	7.0	27.7		13.3		6.6	
Married	35,467	72.4	30.3		19.2		11.1	
Cohabiting	7473	15.3	29.3		15.2		7.4	
Widowed	410	0.8	33.4		24.4		14.9	
Divorced	658	1.3	33.8		17.1		10.5	
Separated	1548	3.2	33.3		18.5		8.2	
**Household characteristics**								
**Drinking water source**				<0.001		<0.001		0.002
Improved	31,445	64.2	29.4		17.4		9.7	
Unimproved	17,523	35.8	31.4		19.5		10.9	
**Toilet facility**				<0.001		<0.001		<0.001
Improved	20,924	42.7	26.7		13.8		7.9	
Unimproved	28,044	57.3	32.7		21.5		11.8	
**Household size**				0.152		<0.001		<0.001
Small	21,256	43.4	29.7		16.6		9.1	
Medium	21,301	43.5	30.2		19.1		10.6	
Large	6411	13.1	31.3		20.5		12.0	
**Frequency of watching television**				<0.001		<0.001		<0.001
Not at all	30,471	62.2	34.4		21.5		11.5	
Less than once a week	6112	12.5	27.0		16.2		9.4	
At least once a week	12,385	25.3	21.2		11.1		7.2	
**Frequency of listening radio**				<0.001		<0.001		<0.001
Not at all	22,380	45.7	33.0		21.0		11.6	
Less than once a week	9596	19.6	29.6		17.9		9.9	
At least once a week	16,992	34.7	26.7		14.7		8.3	
**Frequency of readingnewspaper or magazine**				<0.001		<0.001		<0.001
Not at all	41,619	85.0	31.6		19.8		10.9	
Less than once a week	4364	8.9	23.5		9.8		5.6	
At least once a week	2985	6.1	18.9		7.8		5.9	
**Cooking fuel**				<0.001		<0.001		<0.001
Unclean	44,547	91.0	31.3		19.2		10.6	
Clean	4421	9.0	18.7		8.1		5.5	
**Wealth index**				<0.001		<0.001		<0.001
Poorest	10,900	22.3	36.7		23.7		12.4	
Poorer	10,547	21.5	33.9		20.6		10.4	
Middle	10,023	20.4	30.9		18.6		10.1	
Richer	9362	19.1	27.8		15.3		9.2	
Richest	8136	16.6	18.3		10.5		7.8	
**Contextual-level factors**								
**Place of residence**				<0.001		<0.001		<0.001
Urban	15,775	32.2	22.9		12.4		7.8	
Rural	33,193	67.8	33.6		20.9		11.2	
**Geographical subregions**				<0.001		<0.001		<0.001
Southern	1112	2.3	24.2		9.7		7.4	
Central	9871	20.1	30.2		18.8		10.4	
Eastern	17,903	36.6	33.0		15.0		7.1	
Western	20,083	41.0	27.9		51.2		12.8	

*p*-Values obtained from chi-square test.

**Table 3:** Fixed and random effects result on the association between dietary diversity and stunting among children aged 6–23 months in sub-Saharan Africa.

Variable	Model O	Model I cOR (95% CI)	Model II aOR (95% CI)
**Fixed effect model**			
**Minimum dietary diversity**			
Inadequate		1 (1.00,1.00)	1 (1.00, 1.00)
Adequate		0.86 *** (0.81, 0.91)	0.88 *** (0.83, 0.94)
**Child characteristics**			
**Sex of child**			
Male			1 (1.00, 1.00)
Female			0.63 *** (0.60, 0.66)
**Age of child (months)**			
6–8			1 [1.00, 1.00]
9–11			1.45 *** (1.32, 1.59)
12–17			2.46 *** (2.26, 2.68)
18–23			4.10 *** (3.77, 4.46)
**Size of child at birth**			
Large			1 [1.00, 1.00]
Average			1.37 *** (1.29, 1.45)
Smaller			2.08 *** (1.93, 2.25)
**Maternal characteristics**			
**Maternal educational level**			
No education			1 (1.00, 1.00)
Primary			0.84 *** (0.79, 0.90)
Secondary			0.71 *** (0.66, 0.77)
Higher			0.46 *** (0.36, 0.57)
**Mother’s age (years)**			
15–19			1 (1.00, 1.00)
20–24			0.89 * (0.81, 0.98)
25–29			0.81 *** (0.73, 0.89)
30–34			0.77 *** (0.69, 0.85)
35–39			0.78 *** (0.70, 0.88)
40–44			0.86 * (0.74, 0.99)
45–49			0.78 * (0.62, 0.99)
**Current working status**			
No			1 (1.00, 1.00)
Yes			1.07 ** (1.02, 1.14)
**Marital status**			
Never in union			1 (1.00, 1.00)
Married			0.99 (0.89, 1.10)
Cohabiting			1.02 (0.90, 1.15)
Widowed			0.93 (0.71, 1.22)
Divorced			0.99 (0.78, 1.26)
Separated			1.11 (0.94, 1.31)
**ANC**			
None			1 (1.00, 1.00)
1–3			0.95 (0.87, 1.05)
4 or more			0.80 *** (0.73, 0.88)
**PNC**			
No			1 (1.00, 1.00)
Yes			0.91 *** (0.87, 0.96)
**Frequency of watching television**			
Not at all			1 (1.00, 1.00)
Less than once a week			0.87 ** (0.80, 0.95)
At least once a week			0.82 *** (0.75, 0.90)
**Frequency of reading newspaper/magazine**			
Not at all			1 (1.00, 1.00)
Less than once a week			0.92 (0.83, 1.02)
At least once a week			0.83 * (0.71, 0.97)
**Household factors**			
**Toilet facility**			
Improved			1 (1.00, 1.00)
Unimproved			0.94 (0.88, 1.00)
**Drinking water source**			
Improved			1 (1.00, 1.00)
Unimproved			0.97 (0.92, 1.03)
**Household size**			
Small			1 (1.00, 1.00)
Medium			1.02 (0.96, 1.08)
Large			1.14 ** (1.05, 1.25)
**Cooking fuel**			
Unclean			1 (1.00, 1.00)
Clean			0.92 (0.80, 1.07)
**Wealth index**			
Poorest			1 (1.00, 1.00)
Poorer			0.91 * (0.85, 0.98)
Middle			0.84 *** (0.78, 0.91)
Richer			0.80 *** (0.72, 0.90)
Richest			0.60 *** (0.53, 0.68)
**Contextual-level factors**			
**Place of residence**			
Urban			1 (1.00, 1.00)
Rural			1.05 (0.97, 1.14)
**Geographical subregions**			
Southern			1 (1.00, 1.00)
Central			1.13 (0.93, 1.36)
Eastern			1.20 * (1.00, 1.43)
Western			0.90 (0.75, 1.09)
**Random effect model**			
PSU variance (95% CI)	0.06 (0.05, 0.08)	0.06 (0.05, 0.08)	0.06 (0.04, 0.08)
ICC	0.02	0.02	0.02
Wald chi–square	Reference	26.85 ***	2945.96 ***
**Model fitness**			
Log-likelihood	−29,676.65	−29,655.21	−27,301.93
AIC	59,357.31	59,316.43	54,691.87
N	48,968	48,968	48,968
Number of clusters	1563	1563	1563

Exponentiated coefficients; 95% confidence intervals in brackets; aOR = adjusted odds ratios; CI Confidence Interval; * *p* < 0.05, ** *p* < 0.01, *** *p* < 0.001; 1 = Reference category; MDD = Minimum dietary diversity; ANC = Antenatal care attendance; PNC = Postnatal care; PSU = Primary Sampling Unit; ICC = Intra-Class Correlation; AIC = Akaike’s Information Criterion.

**Table 4:** Fixed and random effects result on the association between minimum dietary diversity and underweight among children aged 6–23 months in sub-Saharan Africa.

Variable	Model O	Model I aOR (95% CI)	Model II aOR (95% CI)
**Fixed effects model**			
**Minimum dietary diversity**			
Inadequate		1 (1.00, 1.00)	1 (1.00, 1.00)
Adequate		0.66 *** (0.61, 0.71)	0.83 *** (0.77, 0.91)
**Child characteristics**			
**Sex of child**			
Male			1 (1.00, 1.00)
Female			0.65 *** (0.61, 0.69)
**Age of child**			
6–8			1 (1.00, 1.00)
9–11			1.36 *** (1.23, 1.51)
12–17			1.46 *** (1.34, 1.60)
18–23			1.66 *** (1.52, 1.81)
**Birth order**			
1			1 (1.00, 1.00)
2–4			0.97 (0.87, 1.07)
5 and above			1.05 (0.91, 1.20)
**Size of child at birth**			
Large			1 (1.00, 1.00)
Average			1.46 *** (1.36, 1.57)
Smaller			2.72 *** (2.48, 2.98)
**Maternal characteristics**			
**Maternal educational level**			
No education			1 (1.00, 1.00)
Primary			0.75 *** (0.69, 0.81)
Secondary			0.61 *** (0.56, 0.67)
Higher			0.49 *** (0.37, 0.66)
**Mother’s age**			
15–19			1 (1.00, 1.00)
20–24			1.00 (0.89, 1.13)
25–29			0.99 (0.87, 1.14)
30–34			1.00 (0.86, 1.17)
35–39			0.96 (0.81, 1.13)
40–44			1.08 (0.89, 1.30)
45–49			0.76 (0.57, 1.02)
**Marital status**			
Never in union			1 (1.00, 1.00)
Married			1.04 (0.91, 1.18)
Cohabiting			0.98 (0.83, 1.15)
Widowed			1.32 (0.97, 1.79)
Divorced			1.02 (0.76, 1.36)
Separated			1.27 * (1.00, 1.60)
**ANC**			
None			1 (1.00, 1.00)
1–3			0.92 (0.84, 1.02)
4 or more			0.81 *** (0.73, 0.89)
**Place of delivery**			
Home			1 (1.00, 1.00)
Health facility			0.77 *** (0.71, 0.83)
Other			1.04 (0.81, 1.33)
**Frequency of watching television**			
Not at all			1 (1.00, 1.00)
Less than once a week			0.87 ** (0.78, 0.96)
At least once a week			0.77 *** (0.70, 0.85)
**Frequency of listening radio**			
Not at all			1 (1.00, 1.00)
Less than once a week			1.00 (0.93, 1.08)
At least once a week			0.94 (0.87, 1.01)
**Frequency of reading newspaper/magazine**			
Not at all			1 (1.00, 1.00)
Less than once a week			0.82 ** (0.72, 0.94)
At least once a week			0.80 * (0.66, 0.98)
**Toilet facility**			
Improved			1 (1.00, 1.00)
Unimproved			1.10 ** (1.03, 1.18)
**Household size**			
Small			1 (1.00, 1.00)
Medium			1.02 (0.95, 1.10)
Large			1.01 (0.91, 1.11)
**Cooking fuel**			
Unclean			1 (1.00, 1.00)
Clean			0.76 ** (0.63, 0.92)
**Contextual-level factors**			
**Place of residence**			
Urban			1 (1.00, 1.00)
Rural			1.15 ** (1.05, 1.25)
**Geographical subregions**			
Southern			1 (1.00, 1.00)
Central			1.46 ** (1.12, 1.91)
Eastern			1.03 (0.79, 1.34)
Western			1.50 ** (1.15, 1.95)
**Random effect model**			
PSU variance (95% CI)	0.74 (0.05, 0.10)	0.07 (0.05, 0.10)	0.07 (0.05, 0.09)
ICC	0.02	0.02	0.02
Wald chi–square	Reference	117.00 ***	2377.44 ***
**Model fitness**			
Log-likelihood	−22,996.44	−22,892.21	−21,316.71
AIC	45,996.88	45,790.42	42,719.42
*N*	48,968	48,968	48,968
Number of clusters	1563	1563	1563

Exponentiated coefficients; 95% confidence intervals in brackets; aOR = adjusted odds ratios; CI Confidence Interval; * *p* < 0.05, ** *p* < 0.01, *** *p* < 0.001; 1 = Reference category; MDD = Minimum dietary diversity; ANC = Antenatal care attendance; PSU = Primary Sampling Unit; ICC = Intra-Class Correlation; AIC = Akaike’s Information Criterion.

**Table 5:** Fixed and random effects result on the association between dietary diversity and wasting among children aged 6–23 months in sub-Saharan Africa.

Variable	Model O	Model I aOR (95% CI)	Model II aOR (95% CI)
**Fixed effect model**			
**Minimum dietary diversity**			
Inadequate		1 (1.00, 1.00)	1 (1.00, 1.00)
Adequate		0.65 *** (0.59, 0.72)	0.87 * (0.78, 0.97)
**Child characteristics**			
**Sex of child**			
Male			1 (1.00, 1.00)
Female			0.68 *** (0.63, 0.73)
**Age of child**			
6–8			1 (1.00, 1.00)
9–11			1.14 * (1.01, 1.28)
12–17			0.93 (0.84, 1.03)
18–23			0.73 *** (0.65, 0.82)
**Birth order**			
1			1 (1.00, 1.00)
2–4			1.05 (0.95, 1.16)
5 and above			1.15 * (1.02, 1.30)
**Size of child at birth**			
Large			1 (1.00, 1.00)
Average			1.32 *** (1.21, 1.45)
Smaller			2.03 *** (1.83, 2.25)
**Maternal characteristics**			
**Maternal educational level**			
No education			1 (1.00, 1.00)
Primary			0.75 *** (0.68, 0.82)
Secondary			0.67 *** (0.60, 0.76)
Higher			0.66 * (0.47, 0.92)
**ANC**			
None			1 (1.00, 1.00)
1–3			0.90 (0.80, 1.02)
4 or more			0.78 *** (0.70, 0.88)
**Place of delivery**			
Home			1 (1.00, 1.00)
Health facility			0.77 *** (0.70, 0.85)
Other			0.65 * (0.44, 0.96)
**PNC**			
No			1 (1.00, 1.00)
Yes			1.05 (0.97, 1.14)
**Current working status**			
No			1 (1.00, 1.00)
Yes			0.84 *** (0.77, 0.91)
**Frequency of watching television**			
Not at all			1 (1.00, 1.00)
Less than once a week			0.91 (0.80, 1.04)
At least once a week			0.82 ** (0.72, 0.93)
**Frequency of listening radio**			
Not at all			1 (1.00, 1.00)
Less than once a week			0.95 (0.86, 1.06)
At least once a week			0.90 * (0.82, 1.00)
**Frequency of reading newspaper/magazine**			
Not at all			1 (1.00, 1.00)
Less than once a week			0.85 (0.70, 1.03)
At least once a week			1.02 (0.80, 1.29)
**Toilet facility**			
Improved			1 (1.00, 1.00)
Unimproved			1.14 ** (1.04, 1.25)
**Household size**			
Small			1 (1.00, 1.00)
Medium			1.00 (0.91, 1.09)
Large			1.00 (0.89, 1.12)
**Cooking fuel**			
Unclean			1 (1.00, 1.00)
Clean			0.72 ** (0.59, 0.89)
**Wealth index**			
Poorest			1 (1.00, 1.00)
Poorer			0.90 (0.81, 1.00)
Middle			0.98 (0.87, 1.10)
Richer			1.05 (0.92, 1.19)
Richest			1.21 * (1.04, 1.41)
**Contextual-level factors**			
**Geographical subregions**			
Southern			1 (1.00, 1.00)
Central			1.02 (0.77, 1.36)
Eastern			0.67 ** (0.51, 0.89)
Western			1.20 (0.91, 1.58)
**Random effect model**			
PSU variance (95% CI)	0.11 (0.08, 0.15)	0.11 (0.08, 0.15)	0.11 (0.08, 0.15)
ICC	0.03	0.03	0.03
Wald chi-square	Reference	63.06 ***	1044.79 ***
**Model fitness**			
Log-likelihood	−15,873.50	−15,808.28	−15,043.51
AIC	31,750.99	31,622.56	30,161.01
N	48,968	48,968	48,968
Number of clusters	1563	1563	1563

Exponentiated coefficients; 95% confidence intervals in brackets; aOR = adjusted odds ratios; CI Confidence Interval; * *p* < 0.05, ** *p* < 0.01, *** *p* < 0.001; 1 = Reference category; MDD = Minimum dietary diversity; ANC = Antenatal care attendance; PNC = Postnatal care PSU = Primary Sampling Unit; ICC = Intra-Class Correlation; AIC = Akaike’s Information Criterion.

## Data Availability

The dataset is available on the following website: https://dhsprogram.com/data/available-datasets.cfm (accessed on 1 August 2021).

## Supplementary Materials

The following are available online at https://www.mdpi.com/article/10.3390/nu13103431/s1, Table S1: Distribution of the types of food groups consumed by the children per country.
