# Supplementary material for: Dietary Diversity and Undernutrition in Children Aged 6–23 Months in Sub-Saharan Africa
Source: Nutrients. 2021 Sep 28;13(10):3431. doi: 10.3390/nu13103431 (PMC8537414; doi:10.3390/nu13103431)
Supplement: Supplementary file 1 [file nutrients-13-03431-s001.zip › nutrients-1377638-supplementary.pdf]

Table S1: Distribution of the types of food groups consumed by the children per country

Countries	Grains, roots and tubers	Legumes and nuts	Dairy products	Flesh foods	Eggs	Vitamin A-rich fruits and vegetables	Other fruits and vegetables
Angola	73.5	22.2	28.7	59.5	14.9	58.7	32.4
Burkina Faso	62.3	7.0	11.8	20.7	5.0	22.7	5.1
Benin	54.3	25.5	30.7	47.8	22.4	31.2	27.1
Burundi	71.5	55.8	23.9	22.3	3.3	91.5	8.1
Congo DR	65.1	11.5	9.6	50.5	9.2	65.9	25.8
Congo	86.5	10.0	49.1	60.2	8.4	59.6	11.8
Cote d'Ivoire	82.3	4.2	15.5	55.6	10.3	17.9	11.0
Cameroon	89.9	13.4	22.1	52.9	14.5	57.2	36.9
Ethiopia	63.9	21.6	38.6	8.5	17.2	28.3	10.5
Gabon	78.9	6.8	71.8	49.2	17.7	38.7	16.8
Ghana	87.5	13.4	22.5	54.1	19.9	41.1	21.7
Gambia	87.6	15.9	36.1	45.5	12.2	22.3	21.7
Guinea	69.2	3.2	24.3	27.1	20.1	37.9	12.3
Kenya	86.7	26.9	57.3	22.9	17.7	66.5	35.4
Comoros	85.9	9.4	35.5	56.0	19.7	46.4	16.8
Liberia	74.5	7.0	15.0	45.5	8.9	39.1	11.5
Lesotho	86.3	18.1	34.1	23.9	25.7	36.4	18.7
Mali	69.9	12.2	28.6	45.6	14.7	43.9	12.9
Malawi	71.2	26.4	10.4	31.1	11.4	73.5	28.5
Nigeria	84.3	35.6	30.8	38.8	17.3	43.4	16.4
Niger	75.5	12.7	16.5	14.7	4.7	27.2	6.5
Namibia	66.4	11.1	33.9	64.9	21.8	41.7	29.8
Rwanda	71.3	65.5	25.5	18.6	4.5	72.1	26.5
Sierra Leone	79.0	18.3	31.9	49.0	18.4	44.3	20.9
Senegal	82.2	9.3	40.9	46.4	10.1	45.0	15.4
Chad	62.1	7.5	25.0	33.6	7.6	24.0	9.4
Togo	82.1	16.5	11.2	55.2	10.9	53.2	10.6
Tanzania	90.9	37.0	23.6	32.7	7.6	65.9	20.2
Uganda	82.2	52.8	29.9	34.3	13.7	50.6	45.1
South Africa	86.1	12.8	72.0	45.8	41.8	43.6	40.3
Zambia	86.4	23.1	12.0	43.4	23.4	64.3	27.6
Zimbabwe	94.4	20.1	20.0	43.1	15.7	60.4	27.1
**All countries**	**76.0**	**21.7**	**27.7**	**38.4**	**14.0**	**48.1**	**20.2**
